# Viewing Ageing Eyes: Diverse Sites of Amyloid Beta Accumulation in the Ageing Mouse Retina and the Up-Regulation of Macrophages

**DOI:** 10.1371/journal.pone.0013127

**Published:** 2010-10-01

**Authors:** Jaimie Hoh Kam, Eva Lenassi, Glen Jeffery

**Affiliations:** 1 Institute of Ophthalmology, University College London, London, United Kingdom; 2 Eye Hospital, University Medical Centre, Ljubljana, Slovenia; University of Nebraska, United States of America

## Abstract

**Background:**

Amyloid beta (Aβ) accumulates in the ageing central nervous system and is associated with a number of age-related diseases, including age-related macular degeneration (AMD) in the eye. AMD is characterised by accumulation of extracellular deposits called drusen in which Aβ is a key constituent. Aβ activates the complement cascade and its deposition is associated with activated macrophages. So far, little is known about the quantitative measurements of Aβ accumulation and definitions of its relative sites of ocular deposition in the normal ageing mouse.

**Methodology/Principal Findings:**

We have traced Aβ accumulation quantitatively in the ageing mouse retina using immunohistochemistry and Western blot analysis. We reveal that it is not only deposited at Bruch's membrane and along blood vessels, but unexpectedly, it also coats photoreceptor outer segments. While Aβ is present at all sites of deposition from 3 months of age, it increases markedly from 6 months onward. Progressive accumulation of deposits on outer segments was confirmed with scanning electron microscopy, revealing age-related changes in their morphology. Such progress of accumulation of Aβ on photoreceptor outer segments with age was also confirmed in human retinae using immunohistochemistry. We also chart the macrophage response to increases in Aβ showing up-regulation in their numbers using both confocal laser imaging of the eye *in vivo* followed by *in vitro* immunostaining. With age macrophages become bloated with cellular debris including Aβ, however, their increasing numbers fail to stop Aβ accumulation.

**Conclusions:**

Increasing Aβ deposition in blood vessels and Bruch's membrane will impact upon retinal perfusion and clearance of cellular waste products from the outer retina, a region of very high metabolic activity. This accumulation of Aβ may contribute to the 30% reduction of photoreceptors found throughout life and the shortening of those that remain. The coating of Aβ on outer segments may also have an impact upon visual function with age.

## Introduction

Age-related macular degeneration (AMD) is the leading cause of blindness in those over 50 years in the Western industrialized world [1]–[3] and is characterized by the formation of drusen, which are extracellular deposits between the retinal pigment epithelium (RPE) and Bruch's membrane [4]–[6]. These deposits result in disturbance in the transepithelial barrier, RPE atrophy, and subsequent degeneration of the neural retina [7]. One of the key constituents of drusen is amyloid beta (Aβ), a protein also present in the brain of Alzheimer's disease (AD) patients [8]–[11]. Aβ causes RPE alterations and dysfunctions leading to retinal degeneration [12]. It is also an activator of the complement cascade and associated with microglia, astrocytes and dendritic cell activation [13]–[16]. The presence of these cells may be indicative of an attempt to clear Aβ [17]–[20], but it has also been suggested that they may play a role in disease development [21]–[23].

Much has been undertaken to study Aβ depositions in the brain of mice models of AD, [24]–[28], however, the retina have received less attention. Three recent studies investigated retinal changes in transgenic AD mouse models. Ning et al. have shown age-dependent Aβ accumulation in the retina, especially in the nerve fibre layer and choriocapillaris, possibly resulting in neurodegeneration [29]. Furthermore, Perez et al. revealed that in the AD mouse retina, there are an age-dependent formation of Aβ plaques, microglial activation, and a functional deficit [30]. Neither observed these changes in age-matched wild-type animals. Dutescu et al., on the other hand, have quantified the amount of amyloid precursor protein (APP) proteolytic products in the retina of transgenic mice models of AD and in normal C57Bl/6/SJL using ELISA and Western blot [31]. In the retina, they have found only trace amounts of Aβ even in transgenic AD mice, indicating minor importance of Aβ in retinal toxicity than suggested by other immunochemical studies.

To date accumulation of the Aβ in the retina has mostly been studied in the transgenic AD murine models to investigate whether toxic Aβ in the retina may cause visual disturbances in patients with AD. However, to our knowledge there is no data on Aβ accumulation in the mouse retina as a part of a normal ageing process. Therefore, our aim was to quantitate the progressive accumulation of Aβ in normal murine ageing retinae using three independent methods to reveal diverse deposition sites and to investigate the relationship between the macrophages and Aβ. We reveal that a key site for Aβ accumulation is on the membrane of the photoreceptor outer segments as well as on blood vessels and Bruch's membrane. We also relate these data with quantitative assessment of macrophage recruitment using both *in vivo* retinal imaging and immunohistochemistry.

## Results

### Imaging and age-dependent accumulation of subretinal macrophages

**Figure 1:**
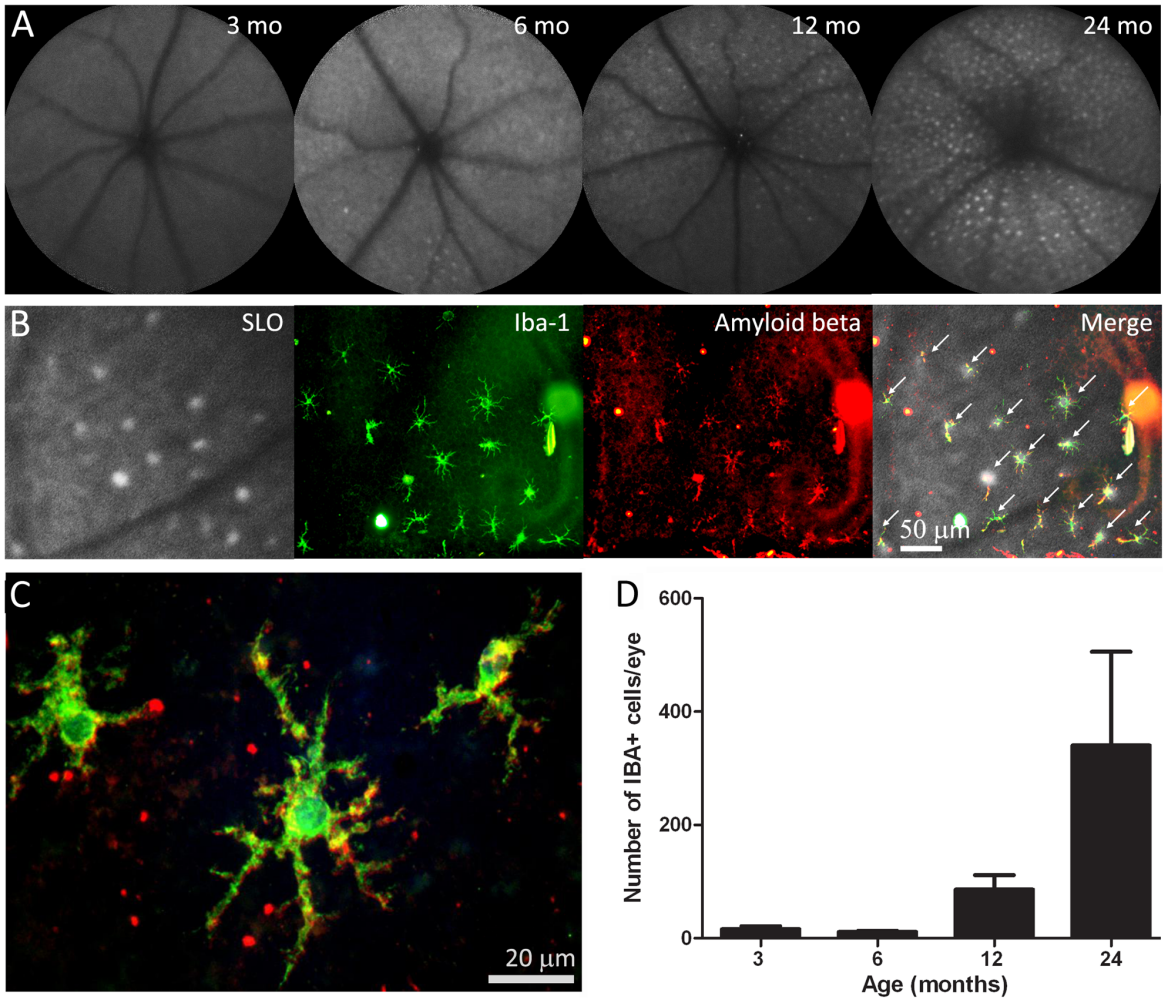
Retinal imaging and macrophage histology. A. Scanning laser ophthalmoscope images of the retinae of mice taken at 3, 6, 12 and 24 months of age. With time there is an accumulation of fluorescent point sources. B. The images are overlaid with the eyecups of a 12 months old mouse once they have been stained for Iba-1 to reveal macrophages and Aβ. This shows that many of the point sources are macrophages containing Aβ (indicated with arrows). These are arranged in a grid like pattern. C. A higher power image of Aβ containing macrophages of a 12 months old mouse. D. The number of macrophages present in the whole mounts at progressive ages. There are significant increases at 12 and 24 months (see text for statistics).

**Figure 2:**
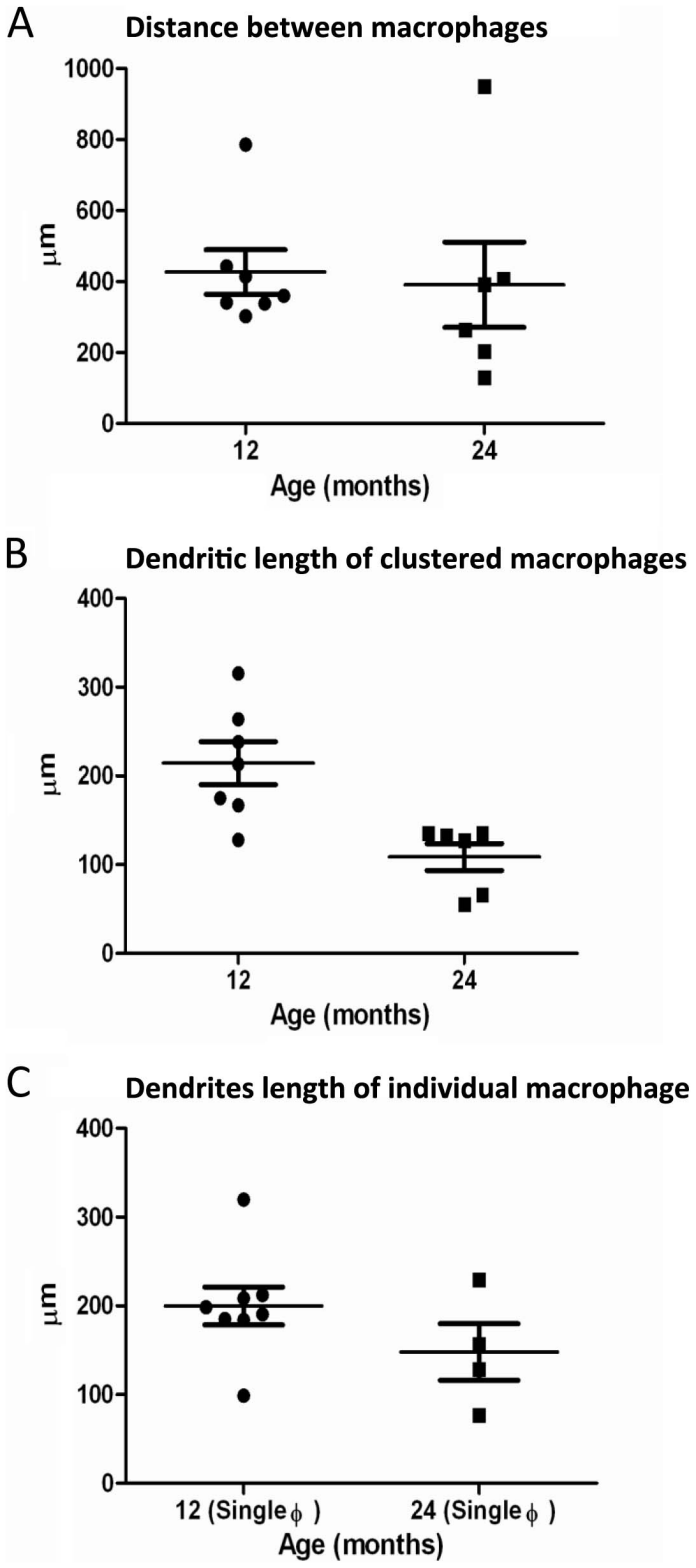
Graphs showing the distance between macrophages and measurement of the dendritic processes. A. Graph showing the distance between macrophages in a cluster of seven cells for the 12 months and six cells for the 24 months old mouse. B. Graph showing the length of the dendritic processes of these seven cells of the 12 months and six cells of the 24 months old mouse (P<0.01). C. Graph showing the length of the dendritic processes of eight individual macrophage cells for the 12 months and four individual cells for the 24 months old mouse.

Fundus autofluorescence images taken using the confocal scanning laser ophthalmoscope (cSLO) at 3, 6, 12 and 24 months revealed an age-dependent increase in the number of hyperautofluorescence spots in sub-retinal regions (Figure 1A). These spots were variable in number but appeared to increase markedly in animals over 6 months of age. When the imaged eyes were subsequently processed histologically for the macrophage marker Iba-1, there was a tight correlation between the hyperautofluorescence sources *in vivo* and the Iba-1 positive cells when the images were overlaid (Figure 1B), hence they are macrophage/microglia cells, a point also noted by Xu, et al. [32]. More that 95% of the Iba-1 positive cells contained Aβ (Figure 1B, Figure 1C). The amount of Aβ in these cells appeared to increase with age. As with the hyperautofluorescence point sources, the number of microglia increased significantly with age (Figure 1D; ANOVA P<0.05). Changes were, however, less marked between 3 and 6 months, but they were significant between these early stages and 12 and 24 months (P<0.05 in both cases). They were also significant between 12 and 24 months (P<0.05). The Iba-1 positive cells were morphologically ramified dendriform and regularly distributed in the sub-retinal space in a mosaic like pattern. The distance between these cells when they were clustered did not change over time but their dentritic processes shortened (P<0.05), perhaps suggesting that they are less efficient in the removal of cellular debris as they cover less surface area (Figure 2A and B). When the Iba-1 positive cells were on their own and not in cluster, the length of the dendritic processes did not change over time (Figure 2C).

### Age-dependent accumulation of Aβ in mouse and human eyes

**Figure 3:**
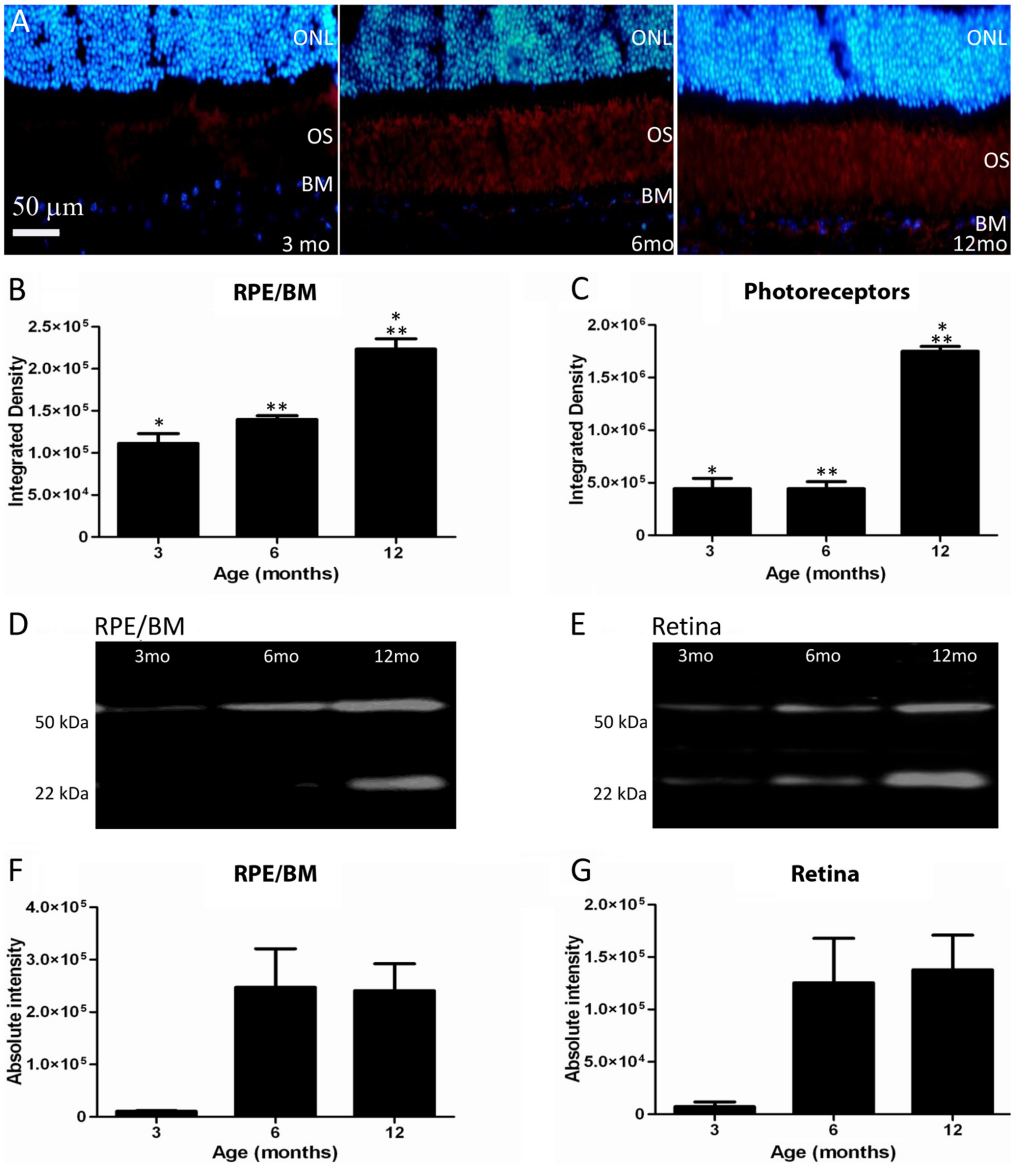
Aβ is deposited at the Bruch's membrane (BM)/RPE interface and among photoreceptor outer segments. A. The accumulation of amyloid beta in sections showing BM/RPE interface and the regions of the outer segments (OS) in mice of 3, 6 and 12 months age. Here Aβ label is red and the outer nuclear layer (ONL) is blue. This progressive accumulation was quantified with two independent methods at the two sites. First, the integrated density of label from immunostained sections was measured. The results of this are shown graphically in B and C. There are significant increases at both sites, particularly at 12 months (see text for levels of significance). Second, Western blots were run for Aβ at each site shown in D and E. F and G show the measurements at the same three time points as in B and C. The amount of Aβ increases significantly over time (see text for levels of significance). Differences between B and C and F and G are probably due to the different amounts of tissue sampled as F and G will also include measures derived from inner and outer retinal blood vessels.

Immunostaining of sections containing the retina and RPE revealed that Aβ expression was primarily present at the level of the photoreceptor outer segment and the Bruch's membrane/RPE interface, along with retinal and choroidal blood vessels. Within these regions Aβ was present even at 3 months of age, and from this point there was a clear age-related accumulation of this material at these sites (Figure 3A). The accumulation of Aβ is presented quantitatively for integrated density at the Bruch's membrane/RPE interface and for the photoreceptor outer segments in immunohistochemical preparations (Figure 3B and 3C) and using Western blot analysis (Figure 3D and 3E). No attempt was made to examine Iba-1 positive cells in section, as only their processes could be commonly identified in such preparations, therefore, reliable counts were not possible.

Aβ deposition on Bruch's membrane/RPE interface increased in stages from 3–6 months and from 6–12 months in immunostained tissue. The difference over the 3 time periods shown in Figure 3B was significant (ANOVA P<0.001). Post-hoc testing revealed that differences between 3 and 12 months and 6 and 12 months were significant (P<0.001; P<0.005, respectively). Similar patterns were found with Aβ deposition over time around photoreceptor outer segments (Figure 3C). Differences in age groups were significant (ANOVA P<0.0001). Post-hoc testing revealed that significant differences were between 3 and 12 months and 6 and 12 months (P<0.0001; P<0.0001 respectively), but not between intermediate stages.

**Figure 4:**
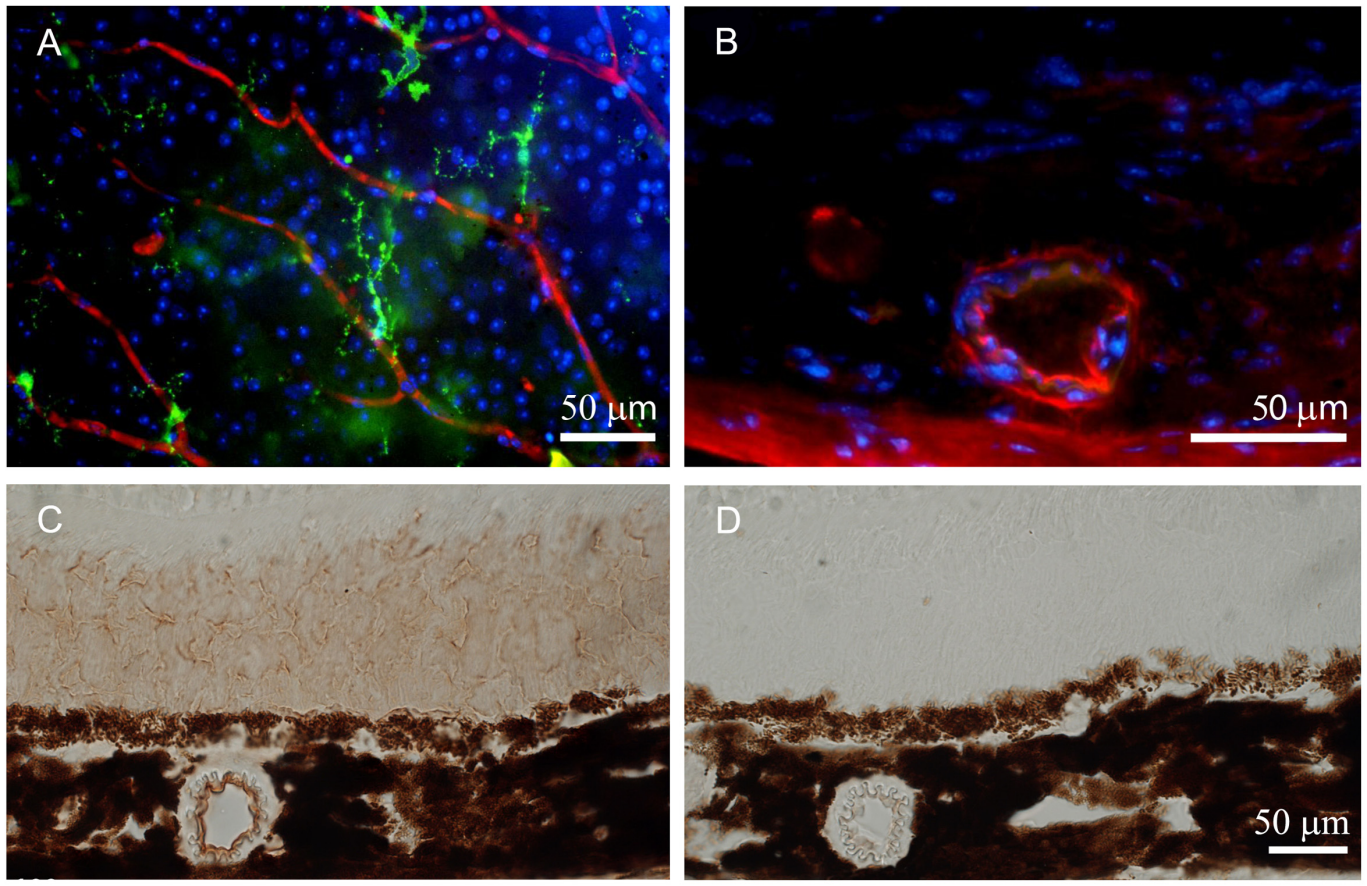
Retinal blood vessels stained for Aβ. A. Inner retinal vessels stained for Aβ (red), Iba-1 (green) and neuronal cell bodies (blue) at 24 months. The amyloid deposits can be seen to be at focal points along the vessel rather than being continuous. B. Choroidal vessels also accumulated Aβ, however, the accumulation of this material appeared to be specific to a sub-group of vessels with other showing no sign of Aβ accumulation. This is taken from a 12 months old animal. C. Immunostaining of retinal section of a 12 months old animal using the colorimetric method (3,3-diaminobenzidine) to confirm the presence of Aβ in the outer segment of the photoreceptor and in the blood vessels. D. Negative control of the colorimetric staining showing the absence of staining.

Aβ staining of whole mounted retinae also revealed the age-dependent accumulation of Aβ in retinal blood vessels. When the tissue was sectioned to examine the accumulation of Aβ in other retinal regions it was clear that it also accumulated in the choroidal blood supply. Here specific vessels became heavily labelled while others appeared to be devoid of Aβ (Figure 4). To confirm the presence of Aβ in the choroidal vasculature, another method of immunohistochemistry was performed using a colorimetric staining to rule out all background staining that might result in false positive staining.

Western blot analysis was also undertaken to quantify age-related changes in Aβ deposition in the mouse RPE/Bruch's membrane interface and in the retina. This showed a significant increase in Aβ with age (Figure 3F and 3G; ANOVA P<0.05). The two most distinct oligomers of Aβ present in the retina and RPE were the hexamers (22–36 kDa) and docecamers (50–64 kDa). In the mouse RPE/Bruch's membrane interface, post-hoc testing demonstrated significant differences between 3 and 6 months and 3 and 12 months (P<0.05; P<0.05 respectively; Figure 3D and 3F). Differences between 6 and 12 months were not significant. In the retina a similar pattern was found (Figure 3E and 3G). Post-hoc testing showed that differences were significant between 3 and 6 months and 3 and 12 months (P<0.05; P<0.01 respectively). These results are largely consistent with that found using immunohistochemistry. The differences at the 6 month stages could be due to the differences in the volumes of the tissue sampled and their origins, as the Western blots included choroidal and retinal blood vessels. If rates of Aβ accumulation in these differed from those in the outer retina and at the Bruch's membrane/RPE interface some variation between immunohistochemical and Western blot analysis might be expected.

**Figure 5:**
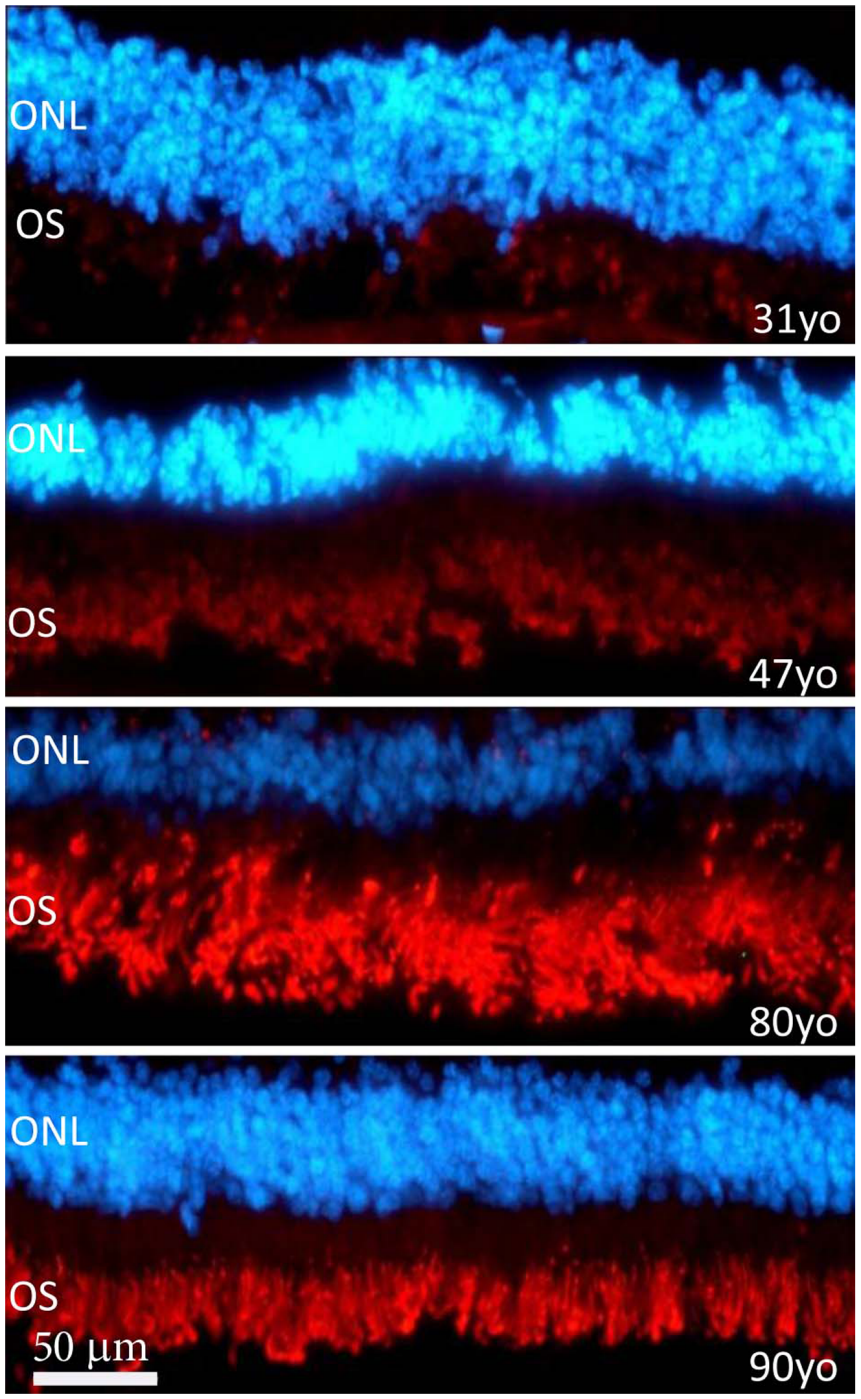
Aβ staining in human outer retina from individuals aged 31, 47, 80 and 90 years. This was undertaken on retinae separated from the RPE. Aβ is red and the outer nuclear layer (ONL) is blue. The outer segments (OS) are positive for Aβ but the intensity of the staining increases with age. In spite of this, the overall progression of Aβ accumulation here mirrors that found in mice.

To determine whether similar patterns of Aβ accumulation were present in photoreceptor outer segments in human tissue, four human retinae spanning 31–90 years were immunostained. We observed an increase of Aβ deposition with age (Figure 5). Although only one retina at each time point was examined with only one method, the results are very similar to that found in mice, with a marked accumulation of Aβ over time. The drop observed in the accumulation of Aβ in the 90 year old retina may be due to age-related loss and shortening of photoreceptors [33].

### Scanning Electron Microscopy imaging of photoreceptor outer segments

**Figure 6:**
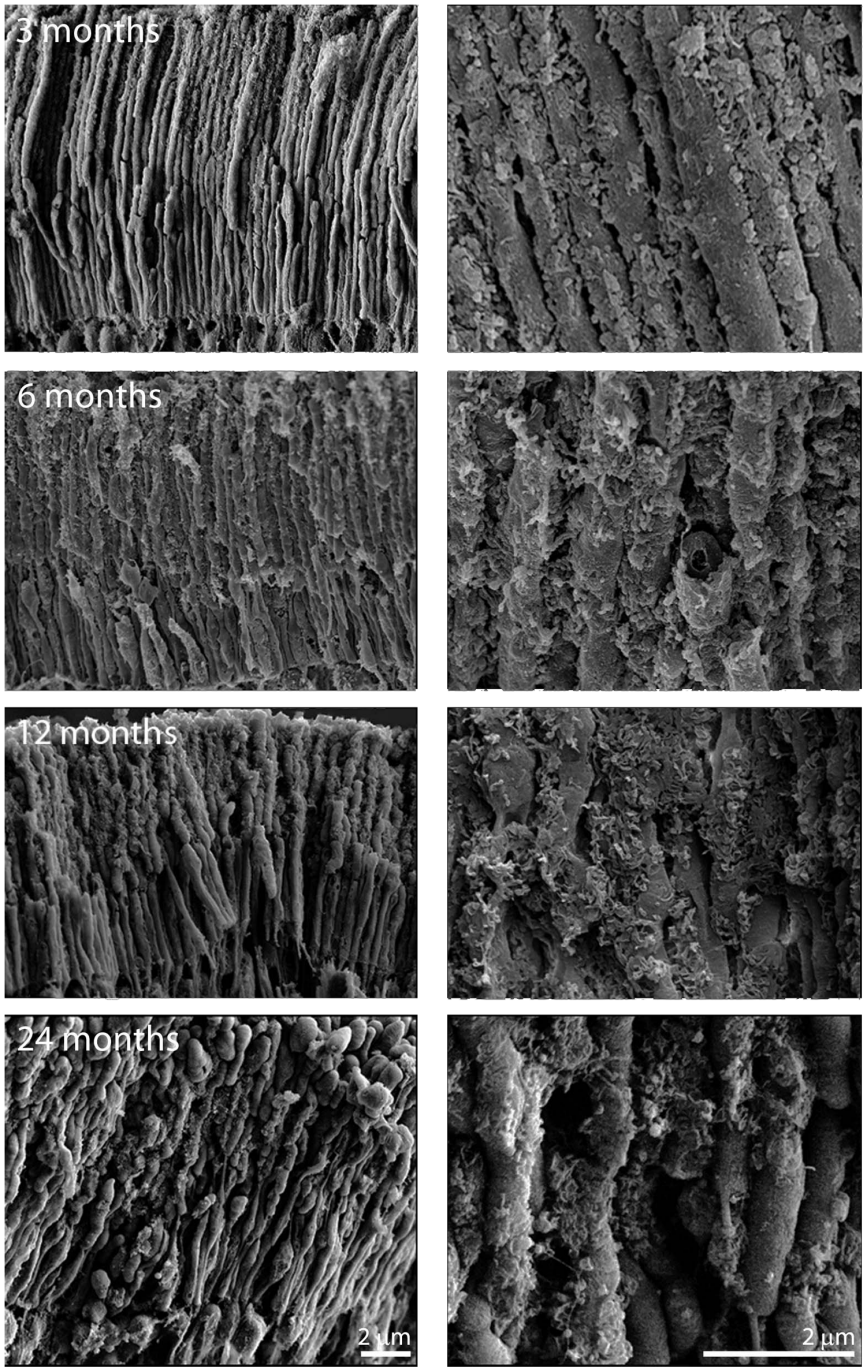
Scanning electron micrographs of photoreceptor outer segments taken from animals at 3, 6, 12 and 24 months of age. In each case the right hand panel is a higher magnification of that on the left and the orientation is such that the RPE would be to the top and the outer nuclear layer to the bottom. Even at 3 months of age deposits can be found on outer segments, however they are more common towards the tip of the outer segment than the base. They are largely spherical in morphology or have rounded edges. By 6 months, their coating has increased and the deposits are present along the length of the outer segment. At 12 months the deposits have thickened, but also appear to have changed qualitatively (See Figure 7). At 24 months while thick deposits remain the tips of many outer segments have enlarged and those that remain are shorter making direct comparison with earlier stages difficult.

The results presented above reveal quantitative increases in Aβ deposition with age in mouse using immunohistochemistry and Western blots. Although these results combine two quantitative methods for measuring Aβ accumulation, the presence of this material was unexpected on photoreceptor outer segments. Consequently, a third method, scanning electron microscopy (EM), was adopted to investigate this deposition in tissue taken at 3, 6, 12 and 24 months (Figure 6). This analysis reveals an increasing accumulation of highly fragmented material on the outer segments with age. This stops at the inner–outer segment junction of the photoreceptor, which reflects the patterns seen using immunohistochemistry.

Deposition can be identified at 3 months and it is more prominent along outer regions of the outer segment at this stage. The amount of material found here increases between 3–6 months (Figure 6). Consistent with the immunostaining (Figure 3C), there was a marked increase in this material at 12 months. At this stage the outer segments appear almost completely wrapped in the material. At 24 months the outer segments appeared qualitatively different as many had enlarged tips (Figure 6). This may be indicative of less efficient patterns of phagocytosis by RPE cells, which remove the end of the photoreceptors daily to compensate for the addition of new photoreceptor disks at the outer segments base [34], [35]. For such reasons, it was not possible to determine whether more debris accumulated on them than at earlier times. At all stages, there is no direct proof that the debris that accumulates on outer segments is Aβ but the close association between immunostaining patterns and the scanning EM images would argue that Aβ was at least an element of such deposits.

**Figure 7:**
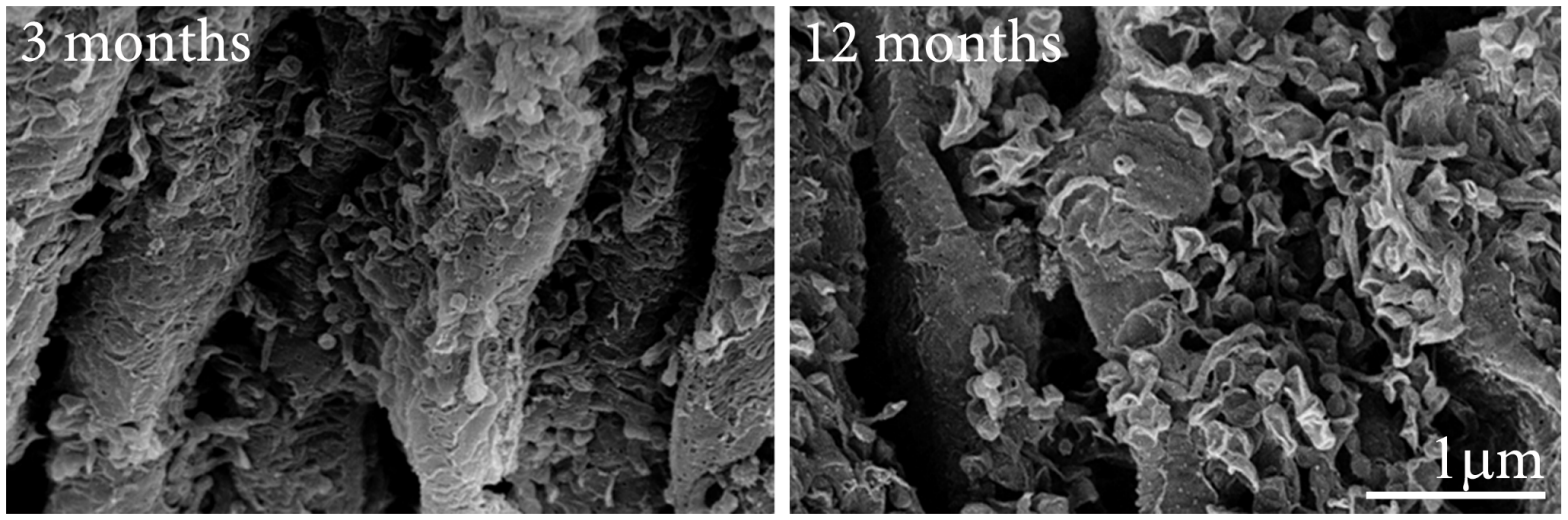
There appeared to be a qualitative change in the morphology of deposits found on outer segments at 12 months of age. The two panels show higher magnification scanning electron micrographs of debris on outer segments at 3 and 12 months. At the earlier stage the deposits are largely spherical with thin processes connecting them to the wall of the outer segment. At 12 months very different picture is present. Here the deposits appear as ruptured hemispheres that have partially collapsed leaving rough edges.

There appeared to be not only a gradual increase in material on outer segments, but also a marked change in its appearance between that found at 3–6 months and that present at 12 months. At the earlier stages, the deposits tend to be spherical or to have rounded edges. At 12 months and after, spherical bodies could also be identified. The majority of the deposits, however, now appeared to have a fragmented wire like appearance. In many cases, the deposits adopted a morphology similar to ruptured spheres, leaving angular edges on the side of a distorted hemisphere (Figure 7).

## Discussion

Aβ is a known constituent of drusen [7], [8], [36], which are age-related deposits found on Bruch's membrane in the human and key risk factors for developing AMD [37]–[39]. In this study, we demonstrate site specific age-related accumulation of Aβ in the normal mouse retina. This occurs primarily among photoreceptor outer segments and on the RPE/Bruch's membrane interface. Two of the independent methods employed are quantitative and Aβ specific (Western blots and immunostaining). The third, the scanning EM, provides structural images for the potential Aβ containing elements deposited on outer segments. We also show *in vitro* and *in vivo* that there is an age-related accumulation of macrophages that internalise Aβ and appear to establish individual exclusion territories. As such, this is the first comprehensive quantitative analysis of age-related Aβ accumulation in the normal murine retina. Further, we extend these studies by examining Aβ deposition on outer segments in the human retinae using immunostaining, showing that as in mice, this material accumulates with age.

Although previous studies have investigated the presence and accumulation of Aβ in the retina, their work was different from ours as they were using transgenic mice models of AD to investigate whether Aβ toxicity could be the cause of visual disturbances in patients with AD. Our main focus here was to investigate the accumulation of Aβ in normal mice as a normal ageing process. We observed an age-related accumulation of Aβ rich extracellular deposits along the Bruch's membrane and also in the outer segments of the photoreceptors in both human and mouse with immunostaining. Western blot results in mouse tissue showed that the most abundant types of Aβ present in the retina and in the RPE-choroidal tissues are the 22–36 kDa and the 50–64 kDa oligomers. These Aβ oligomers were also found in the brain of an AD murine model [24]. In the brain of an AD transgenic mice, it was demonstrated that 56 kDa Aβ oligomers are viable candidates for Aβ assemblies that cause memory deficits as they appear at 6 months old when memory deficit starts. The 27 kDa oligomers are present before memory impairment and they do not affect memory function. Therefore the 50–64 kDa bands obtained in this study may be the one that is toxic in the retina. Lesné et al. have also demonstrated that the level of the 56 kDa remains stable between 6 months and 13 months in the brain and our result reflects the same pattern in our Western blot, as there was no significant difference in the level of Aβ in the mouse retina and at the RPE/Bruch's membrane between these two age groups [24].

Aβ is also deposited in the vascular network of the inner and outer retina. Here the most marked feature was that while inner retinal vessels accumulate Aβ in a patchy progressive manner along their length, choroidal vessels were different in that some accumulate Aβ deposits while others seem to remain Aβ free. There is a correlation between retinal degeneration and AD [29], [31] and it has been demonstrated that AD patients suffer visual disturbances [40]–[42]. One reason for this may be because such disturbances arise from the narrowing of retinal blood vessels and decreased blood flow [43], [44]. Ning et al. [29] have shown that in a transgenic AD murine model, there is an accumulation of Aβ in the retinal and choroidal vasculature, which is consistent with our finding of Aβ deposition in vessels. This may explain the narrowing of the retinal blood vessels and the decreased blood flow found in AD patients.

Accumulation of Aβ in the brain is intimately associated with activated microglial cells and astrocytes. Microglia migrate in response to the chemokine monocyte chemoattractant protein-1 (MCP-1), and cease migration upon interaction with immobilized Aβ [45]. Macrophages incorporate Aβ in an internal attempt to remove it [19], [45]. In the retina, the presence of this incorporated material makes macrophages detectable *in vivo* [46], [47]. Retinal macrophage numbers increase with age in mice [32], [48], however, they become less capable of digesting accumulated waste material and appear overloaded with lipofuscin and Aβ deposits [32]. As a result, more macrophages are probably recruited in an attempt to maintain a homeostasis between the accumulation of cellular debris and its clearance.

In the brain, macrophage numbers have been increased experimentally in a transgenic animal model of AD where amyloid accumulation is marked. This resulted in not only a reduction in amyloid accumulation, but also prevented cognitive decline [49]. It would be surprising if the same were not the case in the retina. Many macrophages seen in older animals appeared bloated with material that included Aβ. As they were so bloated, their ability to remove further quantities of Aβ may have been compromised. As in the brain, one way around this might be the recruitment of new macrophages into the local environment. As we have shown, macrophages in the retina do increase with age, but only over a larger retinal area with no increase in local density. Once initially established, macrophages appear to maintain exclusion territories that restrict the entry of new cells into their matrix. New cells appear to extend the coverage of the matrix but not its local density.

Our study also reveals the presence of extracellular material at the EM level specifically on photoreceptor outer segments. This is a novel finding. The material appeared to initially accumulate at the apical tip of the outer segment and progress down along its length with age, but at no point did this material encroach upon the inner segment. This indicates that the focus of accumulation is at the interface of the RPE with the photoreceptor outer segment tip, implying that this accumulation of debris is in some way related to a decline in the efficiency of the RPE phagocytotic process [34], [35]. If correct, then it would suggest that to fully understand the ageing process in the outer retina, the age-related changes in the RPE population and shifts in their efficiency must be taken into account.

Significant differences were found in the intensity of the Aβ antibody staining on the outer segments around 12 months of age, which coincides with a marked change in the appearance of deposits at the EM level. Initially the deposits were roughly spherical, but later many appeared to rupture giving the appearance of distorted hemispheres with rough sharp edges. The explanation for this distinct morphological change at this stage remains illusive.

Given the large number of EM studies undertaken on the outer retina over the years, it is rather surprising that deposition of debris has not been reported before on outer segments. The answer to this question probably relates to the electron density of the material and the fact that most studies have used relatively young animals with transmission EM. However, the images in this study were generated by platinum coating a fractured surface of the retina which will reveal 3 dimensional deposits on structures. We assume that Aβ is an element of these depositions as there is a close association between the immunostaining pattern and the scanning EM images. An additional consideration is that when Aβ is viewed with transmission EM it tends to be amorphous, and as such is commonly missed unless specifically targeted [50], [51]. Scanning EM has the advantage of providing a 3 dimensional picture where extracellular deposition is much more obvious. Our lab has undertaken transmission EM studies on aged rodent photoreceptors where tissue is viewed in section, and failed to identify the structures seen here [33], although re-examination of the tissue now has revealed amorphous bodies with little structures between the outer segments, particularly around their tips (Unpublished observation).

With ageing many changes occur in the retina. Approximately 25–30% of rod photoreceptors are lost and those that remain shorten by a similar proportion [33], [52]. These changes are probably associated with the distinct functional changes found in the electroretinogram (ERG). Here, the amplitude of component waves of the ERG, both receptoral and post receptoral, decline in magnitude with age [53]–[56]. The outer retina has the largest metabolic demand in the central nervous system [57], [58], and any deposition along Bruch's membrane/RPE interface restricting its access to the choroidal blood supply is likely to be detrimental to retinal function, probably contributing to age-related cell loss. More intriguing, is the potential impact the outer segment debris is likely to have upon photoreceptor function, which is unknown.

## Materials and Methods

### Ethics Statement

All animals were used with University College London ethics committee approval and under a UK Home Office project licence (PPL 70/6571). All animal procedures conformed to the United Kingdom Animal License Act 1986 (UK). Human eyes were obtained from the eye bank at Moorfields Eye Hospital with the approval of Moorfields and Whittington Research Ethics Committee (06/Q0504/78).

### Animals and experimental paradigms

C57 BL/6 mice were housed in a temperature controlled environment with a 12 hour day (160 lux) light/dark cycle. Three groups of C57BL/6 mice were used, each containing 8 animals at 3, 6, 12 months (16 eyes per group). An additional 4 animals were used at 24 months of age (8 eyes). Hence, 28 animals were used in total. Both eyes were used from each animal. The 3, 6, and 12 months old animals were divided between 5 experiments. The first was the non-invasive imaging of the outer retina/RPE undertaken on the right eye prior to sacrifice. Four eyes from each group were then used for cryosectioning and antibody staining. Four were used for confocal SEM, four were taken for Western blot analysis and four were used for whole mounts of the RPE surface. The outer retina and Bruch's membrane/RPE interface were the targeted areas of interest because this is a key area for accumulation of age-related deposits [37]–[39], [59]. The 24 months old animals were only used for retinal imaging followed by antibody staining of whole mounts of the sub-retinal space and SEM.

### In vivo imaging

Mice were anaesthetized (6% Ketamine, (Fort Dodge, UK) 10% Dormitor, (Pfizer, UK) and 84% sterile water at 5 ul/g intraperitoneal injection) and their pupils were dilated (1% tropicamide, Bausch and Lomb, UK). Fundus photographs were taken with a digital camera mounted on a modified cSLO (Heidelberg Retina Angiograph, Heidelberg Engineering, Germany) where the pinhole diameter had been reduced to 100 µm to improve axial resolution, and the laser power increased to improve signal-to-noise ratio. Power at the mouse pupil was measured to be 1400 µW at 488 nm.

### Immunohistochemistry

All mice were sacrificed by exposure to CO_2_. The eyes were removed and fixed in 4% paraformaldehyde in phosphate buffered saline (PBS), pH 7.4 for 1 h. They were then cryo-preserved in 30% sucrose and embedded in optimal cutting temperature compound (OCT, Agar Scientific UK). Cryostat sections were cut at 10 µm and thaw-mounted onto charged slides. Immunohistochemistry was performed at room temperature to reveal Aβ on the left sectioned eye and for Aβ and microglia Iba-1 on the right eye which was processed as a whole mount after cSLO imaging.

Sections were incubated for 1 h in 5% Normal Donkey serum in 0.3% Triton X-100 in PBS, pH 7.4, followed by an overnight incubation with the following primary antibodies; mouse monoclonal antibody to Aβ 4G8 (1∶500, Covance, UK) and rabbit polyclonal antibody to Iba-1 (1∶1000, A. Menarini Diagnostics, UK). Primary antibodies were made in 1% Normal Donkey Serum in 0.3% Triton X-100 in PBS. After Primary antibody incubation, sections were washed three times in 0.1 M PBS and then incubated in respective secondary antibodies conjugated with either Alexa fluor 488 or 568 (Invitrogen Molecular Probes, UK), made up in 2% Normal Donkey Serum in 0.3% Triton X-100 in PBS at a dilution of 1∶2000. These were added to the sections and incubated for 1 h. In negative controls the primary antibody was omitted. After secondary incubation sections were washed several times and the nuclei stained with 4′, 6-diamidino-2-phenylindole (Sigma Aldrich, UK) for 1 min. Slides were then washed 3 times in PBS and several times in Tris buffered Saline (pH 7.5). Slides were mounted in Vectashield (VECTOR Laboratories, UK) and coverslipped. Sections were viewed and images captured using Epi-fluorescence and bright-field. 24-bit colour images were captured at 3840×3072 pixel resolution.

Retinal sections of 12 months old mice were processed for immunohistochemistry using the streptavidin horse radish peroxidase complex. The sections of the eye were incubated for 1 hour in a 5% Normal Donkey serum in 0.3% Triton X-100 in PBS, pH 7.4, followed by an overnight incubation with the mouse monoclonal antibody to amyloid beta which was made in 1% Normal Donkey Serum in 0.3% Triton X-100 in PBS. After the primary antibody incubation, the sections were washed three times in 0.1 M PBS and then treated with 0.3% hydrogen peroxide in PBS to quench endogenous peroxidase activity. After several washes, the tissues were incubated with a biotin–SP conjugated secondary antibody against mouse (Jackson ImmunoResearch Laboratories, 1∶1000) which were made up in 2% Normal Donkey Serum in 0.3% Triton X-100 in PBS, were added to the sections and incubated for 1 hour at room temperature. Negative controls were done by omitting the primary antibody. After the secondary antibody incubation, the sections were washed several times and then incubated in a ready to use horseradish peroxidise Streptavidin solution (VECTOR Laboratories, UK) for 30 minutes, followed by a peroxidase substrate solution, 3,3-diaminobenzidine (DAB) for 1 minute. Slides were mounted in Vectashield (VECTOR Laboratories, UK) and coverslipped after several washes in PBS and TBS. Sections were viewed and images captured using an Epi-fluorescence bright-field microscope (Olympus BX50F4, Olympus, Japan), where data were captured as 24-bit colour images at 3840×3072 pixel resolution using Nikon DXM1200 (Nikon, Tokyo, Japan) digital camera.

For whole mounts, eyes were fixed as above and washed with PBS directly after cSLO imaging. The cornea, lens and retina were removed to expose the sub-retinal space. To flatten these preparations, 5–6 radial cuts were made in each. After several washes with PBS, the RPE-choroidal tissues were blocked and permeabilised with 5% Normal Donkey serum with 3% (v/v) Triton X-100 in PBS for 2 h. These were incubated overnight in two primary antibodies: mouse monoclonal to Aβ beta 4G8 (1∶500, Covance, UK) and rabbit polyclonal antibody to Iba-1 (1∶1000, A. Menarini Diagnostics, UK) in 1% Normal Donkey Serum in 3% Triton X-100 in 0.1 M PBS. Preparations were washed 3 times in PBS and incubated in respective secondary antibodies made up in 2% Normal Donkey Serum in 0.3% Triton X-100 in PBS at a dilution of 1∶2000, and incubated for 2 h at room temperature. After secondary antibody incubation, preparations were washed several times and the nuclei stained as above. The RPE-choroidal tissues were then washed 3 times in 0.1 M PBS and again in several washes in Tris buffered Saline (pH 7.5) and mounted, coverslipped and imaged as above.

Four human eyes were fixed in 10% formalin for at least 24 hours and then dissected whereby the lens, the sclera and the RPE were removed and the retina were cut into smaller pieces. After several washes in PBS, the retinae were cryoprotect in sucrose and then embedded in OCT. 10 µm sections were made and thaw-mounted onto charged slides. Immunohistochemistry was performed at room temperature in the same way as it was done with the mice eye sections.

The mouse monoclonal antibody to Aβ 4G8 which was used in immunohistochemistry and Western blot, is specific for the Aβ ectodomain (amino acid sequence 17–24 in human), a sequence that does not overlap with that of secreted APP and is identical in human, mouse and rat. Therefore this antibody excludes the possibility that the protein expression observed in the immunohistochemistry and the bands obtained in the Western blot were degradation products of soluble APP which lacks the Aβ ectodomain (Aβ_17–24_) [24].

### Scanning Electron Microscopy

Retinae were fixed in 2% paraformaldehyde and 2% glutaldehyde in PBS for 24 h, followed by washing in PBS and then post fixed in 1% osmium tetroxide in 0.1 M PBS for 2 h. Tissues were then thoroughly washed in distilled water and dehydrated through a graded series of ethanol. The specimens were dried with a critical dry point apparatus. After which they were coated with platinum and analysed using a Carl Zeiss scanning electron microscope.

### Western blot analysis

The eyes were dissected on ice and the retina and RPE-choroidal tissues separated and frozen in liquid nitrogen and stored at −80°C The retina and RPE-choroidal tissues were sequentially extracted. The samples were homogenized in 2% sodium dodecyl sulfate with protease inhibitor cocktail (Roche diagnostics), then centrifuged at 13,000× g. The supernatant was then transferred to a new microcentrifuge tube and the resultant pellet extracted with 70% formic acid in water. It was centrifuged at 13,000× g and the supernatant transferred to the microcentrifuge tube and the pellet discarded. The formic acid in the supernatant was evaporated using a speed-Vac concentrator (The Eppendorf Vacuum Concentrator, Brinkmann, USA) and the protein pellet was reconstituted in 10% dimethyl sulfoxide in 2 mol/L Tris-HCl. The protein concentration was measured with an absorbance of 450 nm. Equal amounts of proteins were separated by a 10% sodium dodecyl sulfate-polyacrylamide gel electrophoresis and electrophoretically transferred onto nylon membranes. The membranes containing the transferred proteins were pre-treated with 5% non-fat dried milk in 1 M PBS (pH 7.4) overnight and incubated for 1 h with monoclonal Aβ antibody (1∶1000, Covance) followed by several washes in 0.05% Tween-20 in 1 M PBS. The membranes were then incubated with a goat anti-mouse IgG peroxidise conjugated secondary antibody (1∶10 000, Thermo Scientific) for 1 h. Aβ immunoreactivity was visualized by exposing x-ray film to blots incubated with ECL reagent (SuperSignal West Pico, Thermo Scientific, UK). Total protein profile was determined by gel staining with Coomassie Blue to check that protein extraction was consistent.

### Analysis

#### Measurement of the distance between macrophages

Images of clustered and individual macrophages for 12 and 24 months were captured using a 40X objective lens and a 10X eyepieces in JPEG format using Epi-fluorescence bright-field with a 24-bit colour images at 3840×3072 pixel resolution using Nikon DXM1200 (Nikon, Tokyo, Japan) digital camera. Images were put together using Adobe Photoshop CS4 and the dendrite's length was measured from the centre of the nucleus of the macrophage to the end of dendrites. To determine whether macrophages were regularly spaced, small clusters of six cells were photographed as above and the distances between the nucleus of each cell and its nearest neighbour were measured.

#### Counting of macrophages

Images were captured using a 20X objective lens and a 10X eyepieces in JPEG format using the Epi-fluorescence bright-field microscope with a 24-bit colour images at 3840×3072 pixel resolution using a Nikon DXM1200 digital camera. The images were then put together by Adobe Photoshop CS4 extended. The iba-1 positive cells were counted using the count tool.

#### Measurement of Aβ in Bruch's membrane/RPE interface and photoreceptor in immunostaining

Fluorescence images of the area around the optic nerve head were taken in JPEG format using a 40X objective lens and a 10X eyepieces, using an Epi-fluorescence bright-field microscope (Olympus BX50F4, Olympus, Japan) with a 24-bit colour images at 3840×3072 pixel resolution using Nikon DXM1200 (Nikon, Tokyo, Japan) digital camera. The pictures were put together and the integrated density which is the product of the area chosen (in pixels) and the mean gray value (the measurement of the brightness) were measured using Adobe Photoshop CS4 extended. The lasso tool was used to draw a line all the way around the Bruch's membrane and the integrated density was measured. The same goes for the outer segments whereby the line was drawn around the outer segments of the photoreceptor.

#### Measurement of Aβ in RPE-choroidal and retina in Western blot

The scanned pictures of the protein gel were inverted to grayscale format and the mean gray value was measured for each protein band by using the lasso tool to draw a line all the way around the edges of the band using Adobe Photoshop CS4 extended. The absolute intensity was calculated by multiplying the mean gray value and the pixel value.
